# Ultrastructural Changes in Autopsy Tissues of COVID-19 Patients

**DOI:** 10.7759/cureus.31932

**Published:** 2022-11-27

**Authors:** Aasma Nalwa, Vikarn Vishwajeet, Deepak Kumar, Abhishek Purohit, Mayank Garg, Dr. Tanuj Kanchan, Naveen Dutt, Nikhil Kothari, Suryanarayanan Bhaskar, Poonam Elhence, Pradeep Bhatia, Vijaya L Nag, Mahendra Kumar Garg, Sanjeev Misra, Alok Pandey, Alok Dhawan

**Affiliations:** 1 Pathology and Lab Medicine, All India Institute of Medical Sciences, Jodhpur, IND; 2 Pathology, All India Institute of Medical Sciences, Jodhpur, Jodhpur, IND; 3 Histopathology, Sawai Man Singh (SMS) Medical College, Jaipur, IND; 4 Medicine, All India Institute of Medical Sciences, Jodhpur, IND; 5 Pathology and Hematopathology, All India Institute of Medical Sciences, Jodhpur, IND; 6 Neurological Surgery, All India Institute of Medical Sciences, Jodhpur, IND; 7 Forensic Medicine and Toxicology, All India Institute of Medical Sciences, Jodhpur, IND; 8 Pulmonary and Critical Care Medicine, All India Institute of Medical Sciences, Jodhpur, IND; 9 Anesthesiology and Critical Care, All India Institute of Medical Sciences, Jodhpur, IND; 10 Pathology, All India Institute of Medical Sciences, Jodhpur, IND; 11 Anesthesiology, All India Institute of Medical Sciences, Jodhpur, IND; 12 Microbiology, All India Institute of Medical Sciences, Jodhpur, IND; 13 Surgical Oncology, All India Institute of Medical Sciences, Jodhpur, IND; 14 Nanomaterial Toxicology Laboratory, Council of Scientific & Industrial Research (CSIR) - Indian Institute of Toxicology Research, Lucknow, IND; 15 Research and Development, Centre for Biomedical Research, Lucknow, IND

**Keywords:** lung, autopsy, immunohistochemistry, transmission electron microscopy, covid-19

## Abstract

Introduction: The COVID-19 pandemic resulted in substantial morbidity and mortality across the world. The prognosis was found to be poor in patients with co-morbidities such as diabetes, hypertension, interstitial lung disease, etc. Although biochemical studies were done in patient samples, no study has been reported from the Indian subcontinent about ultrastructural changes in the vital organs of COVID-19 patients. The present study was, therefore, conducted to understand the ultrastructural changes in the lung, liver, and brain of the deceased patients.

Methods: The present study was conducted on samples obtained from reverse transcription-polymerase chain reaction (RT-PCR)-positive patients who were admitted to a tertiary care hospital in Western India. Core needle biopsies were done in eight fatal cases of COVID-19. The samples were taken from the lungs, liver, and brain and subjected to light microscopy, immunohistochemistry (IHC), and transmission electron microscopy (TEM). Clinical details and biochemical findings were also collected.

Results: The study participants included seven males and one female. The presenting complaints included fever, breathlessness, and cough. Light microscopy revealed diffuse alveolar damage in the lungs. Further, a positive expression of SARS-CoV-2 nucleocapsid protein was observed in the pulmonary parenchyma of five patients. Also, the TEM microphotograph showed viral particles of size up to 80nm localized in alveolar epithelial cells. However, no viral particles were found in liver or brain samples. In the liver, macrovesicular steatosis and centrizonal congestion with loss of hepatocytes were observed in light microscopy.

Conclusion: This is the first study in the Indian population showing the *in-situ* presence of viral particles in core biopsies from fatal cases of COVID-19. As evident from the results, histology and ultrastructural changes in the lung correlated with the presence of viral particles. The study revealed a positive correlation between the damage in the lungs and the presence of viral particles.

## Introduction

The COVID-19 pandemic resulted in substantial morbidity and mortality across the world with over six million deaths [1]. Although the pandemic reportedly started in China in late 2019, it spread around the world within a few months. India reported its first case from Kerala on 27th January 2020 [2]. Since then, in India, 44 million cases have been reported of which 0.526 million died [3].

There were no global guidelines for the management of COVID-19, and in most cases, the management varied with the severity of the illness. Significant heterogeneity was observed in the outcomes of these patients [4]. Several studies have shown that the cycle threshold (Ct) in reverse transcription-polymerase chain reaction (RT-PCR) is inversely correlated with the viral load and disease outcomes [5,6]. Magleby et al., in 2021, reported that a low Ct value (less than 25) had a three-time increased risk of intubation and a five to six-time increased risk of mortality [7]. However, this finding was not consistently noted. Camargo et al., in 2021, observed no differences in the initial Ct values between survivors and non‐survivors or mild/moderate versus severe/critical illness at the maximum point of illness [8].

Further, inflammatory and biochemical parameters have been used to predict disease outcomes [9,10]. The C-Reactive protein (CRP; >10 mg/L) and procalcitonin ( >0.5 ng/ml) are predictors of poor prognosis [11]. Also, higher baseline IL-6 levels correlated with the severity of illness, bilateral lung involvement, and mortality [12]. Oussalah et al., in 2020, used a large dataset of 59 biochemical parameters to estimate the occurrence of organ dysfunction and severity of inflammatory response and their association with acute respiratory failure and death. The authors reported that there is no association between these markers and the risk of death [13]. There exists a gap in the existing studies regarding the underlying mechanism of the disease.

Histological changes in various organs in autopsy studies have been reported [14-18]. A few have identified the virus using different orthogonal approaches such as transmission electron microscopy (TEM), immunohistochemistry (IHC), RNA in-situ hybridization, and polymerase chain reaction [17,19-23]. When performed in autopsied tissue samples, TEM offers a unique opportunity to visualize the viral particles in different organs directly, thus aiding in determining SARS-CoV-2 distribution and cellular location. This would be instrumental in elucidating the pathogenesis of COVID-19 infections. Most of these studies have been conducted outside India, and only a few describing histological changes in different tissues have been reported from the Indian population [18,24-26]. The most affected organ was the lung and, the characteristic histological change reported in COVID-19 was diffuse alveolar damage. This was often accompanied by a variable degree of interstitial fibrosis, squamous metaplasia, multinucleation, and vascular microthrombi [14-18,27]. A few in-vitro studies from India focussed on TEM imaging of the SARS-CoV-2 virus in culture [28-30]. However, there is no study on the Indian population using TEM in autopsy tissue samples. The present study was aimed at identifying the presence of viral particles in autopsy samples obtained from the lungs, liver, and brain using IHC and TEM. Also, histological changes in these organs were observed using light microscopy.

## Materials and methods

Materials

Immunohistochemistry was performed for the SARS-CoV-2 nucleocapsid protein (Novus Biological, NB100-56576, 1:300 dilution). Sodium cacodylate, osmium tetraoxide, and glutaraldehyde were purchased from Sigma-Aldrich (St. Louis, MO, USA). Dodecenyl Succinic Anhydride (DDSA) and 2,4,6 Tri-dimethylaminomethyl phenol (DMP-30) were purchased from Ladd’s Research (Williston, VT, USA). Uranyl acetate and lead citrate were purchased from Electron Microscopy Sciences (Hatfield, PA, USA). All other chemicals were purchased locally and were of analytical reagent grade.

Methods

The present study was conducted on samples obtained from RT-PCR-positive patients who were admitted to a tertiary care hospital in Western India. These samples were obtained from April 2020 to December 2020, which corresponds to the first wave of COVID-19 in India. Exclusion criteria included cases where samples could not be taken within six hours of death and cases where consent could not be obtained. Sample size calculation was not done. Samples were taken from eight cases of fatal COVID-19, in which the relative of the deceased had given informed consent. Clearance from the institutional ethics committee was also taken (approval no.: AIIMS/IEC/2020/3189).

Autopsy procedure

In the COVID-19 cases, open post-mortem autopsies were not performed due to the high risk involved. Therefore, samples of the lungs, liver, and brain from the deceased were obtained using core needle biopsies. Biopsies from each lung were taken with the aid of an antemortem chest radiograph from the most affected lobes. Core biopsies from the liver were taken blindly, while biopsies of the frontal lobe of the brain were obtained using a small-bore needle. The samples were immediately placed in 10% neutral buffered formalin (for light microscopy and IHC) and 2.5% glutaraldehyde (for TEM).

Light microscopy

Samples fixed for 48 hours in 10% neutral buffered formalin were processed and the slides were prepared. The tissue sections were cut at 3µm thickness and staining was done as per the standard protocol. For hematoxylin and eosin staining, the slides were rehydrated through descending grades of alcohol to water. It was stained in alum hematoxylin for 20 minutes, followed by bluing in running tap water. The slides were then stained in 1% eosin for 10 minutes. Following this, the slides were washed in running tap water, dehydrated, and mounted. Additional stains (periodic acid-Schiff, reticulin, Masson's trichrome, and phosphotungstic acid hematoxylin) were used.

Immunohistochemical studies

Immunohistochemistry was performed for SARS-CoV-2 nucleocapsid protein (Novus Biological, NB100-56576, 1:300 dilution) in all cases and on all three organs. Pressure cooker antigen retrieval was performed in citrate buffer.

Transmission electron microscopy

The TEM was performed in six cases for three organs: lungs, liver, and brain. For TEM, two to four 2mm^3^ pieces of lung, liver, and brain were cut and immersed in 2.5% glutaraldehyde vials separately. The sample was then transported to one of the reference centers of Central India within 24 hours while maintaining the cold chain. Once these TEM samples were received, all the samples were washed three times, each for 15 minutes, with 0.1 M sodium cacodylate buffer followed by adding 1% osmium tetraoxide (OsO_4_) to each sample. These samples were then stored at 4℃. The next day, the samples were washed with 0.1M sodium cacodylate buffer to remove OsO_4_. The samples were completely dehydrated using an ascending concentration of acetone. The samples were placed in the embedding media, which was prepared using Araldite 502, DDSA, and DMP-30. The sections were cut and stained with uranyl acetate and lead citrate and examined on TecnaiTM G2 Spirit (Field Electron and Ion Company, OR, USA).

Patient details

For all the cases, demographic details such as age and sex as well as comorbidities, presenting complaints, duration of hospital stay, duration of symptom onset to death, and results of laboratory investigations (including complete blood count (CBC), D-dimer, kidney function tests (KFTs), liver function tests (LFTs)) were taken from medical records and compiled.

## Results

Clinical and laboratory findings

The results showed a clear distinction between the changes observed in the lungs by comparison to that in the liver and brain. As evident from Table 1, the mean age of the cases was 68.5 years (range: 44 to 75 years) and seven out of eight cases were males. The mean duration from onset of symptoms to death was 10.7 days (range: five to 15; Table 1). The presenting complaints included shortness of breath (8/8, 100%), fever (6/8, 75%), and cough (4/8, 50%). Co-morbidities were noted in six cases, of which two patients had two co-morbidities. Type 2 diabetes mellitus was noted in three patients, hypertension in two patients, and obesity in two patients. One patient had an intraventricular hemorrhage. All patients were on the ventilator at the time of death.

**Table 1:** Demographic and clinical findings of COVID-19 patients DM: Diabetes mellitus, HTN: Hypertension, IHD: Ischemic heart disease, IVH: Intraventricular hemorrhage; SOB: Shortness of breath

Patient #	1	2	3	4	5	6	7	8
Age (in years)	67	55	74	73	51	44	75	70
Gender	Male	Male	Female	Male	Male	Male	Male	Male
Symptoms onset to death (days)	5	12	10	14	15	12	8	10
Presenting complaints	Fever, cough, SOB	Cough, SOB	Fever, cough, SOB	Fever, SOB	Fever, SOB, orthopnoea	Cough, SOB	Fever, SOB	Fever, cough, SOB
Co-morbidities	Obesity	DM	DM, HTN	-	IHD	IVH	-	DM, HTN
Ventilator	+	+	+	+	+	+	+	+

Haemogram, blood coagulation, serum inflammatory markers, liver function test (LFT), and kidney function test (KFT) are summarized in Table 2. Four cases exhibited mild anemia while three had leucocytosis and one showed thrombocytopenia (Table 2). Liver function tests and kidney function tests were deranged in six (75%) and three (37.5%) cases, respectively.

**Table 2:** Detailed laboratory findings of COVID-19 patients Hb: Hemoglobin, TLC: Total leucocyte count, Plts: Platelets, AST: Aspartate transaminase, ALT: Alanine transaminase, ALP: Alkaline phosphatase, Sr Cr: Serum creatinine, APTT: Activated partial thromboplastin time, INR: International normalized ratio, CRP: C-reactive protein, IL-6- Interleukin 6, NA: Not available

Patient #	1	2	3	4	5	6	7	8
Hb (g/dl)	11.9	12.4	9.5	12.5	15.1	10.2	10.5	13.3
TLC (10^3^/ul)	8	11	8.6	15.3	17	17.6	5.8	9.5
Plts (10^3^/ul)	304	233	272	349	244	30	226	190
AST (IU/L)	41.6	62	31.4	36.9	80.3	238	64.3	70.8
ALT (IU/L)	18.4	27.1	14.2	15.1	72.5	292	86.3	29.1
ALP (IU/L)	43	54	150	89	98	54	88	117
Sr Cr (mg/dl)	1.04	1.02	0.71	1.33	0.82	1.97	0.87	2.25
Sr Urea (mg/dl)	33	46	16	29	65	195	48	86
D-dimer (ug/ml)	0.36	NA	NA	0.85	6.48	1.31	2.73	NA
APTT (sec)	47.6	NA	41.6	49.4	29.0	17.5	26.1	NA
INR	1.0	NA	1.17	NA	1.01	1.2	1.35	NA
Ferritin (ng/ml)	289.2	NA	77.37	NA	NA	3503	2589	1273
CRP (mg/l)	141.57	73.21	108.5	NA	86.74	NA	NA	NA
IL-6 (ng/ml)	NA	NA	1.2	NA	3.9	17.3	163.2	NA

Histopathological findings

**Figure 1:**
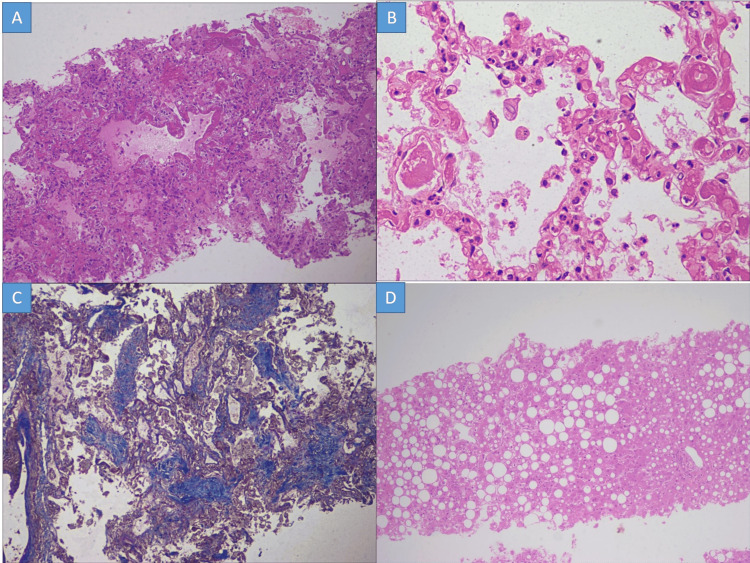
Histopathological changes in the lung A: Deep eosinophilic hyaline membrane along the alveolar lining featuring the exudative phase of diffuse alveolar damage (H&E stain x100); B: Presence of microthrombi in small interalveolar septal capillaries (H&E stain x400); C: Masson trichrome highlights loose fibroblastic plug in the alveolar spaces consistent with organizing phase of diffuse alveolar damage (Masson trichrome stain x100); D: Diffuse macrovesicular steatosis with foci of neutrophilic lobular inflammation (H&E stain x100) H&E: Hematoxylin and eosin

Marked histopathological changes observed in the lung are summarized in Table 3 and Figure 1 (A to C). There was a deposition of deep eosinophilic membrane along the alveolar lining and distal bronchioles, which was positive for PAS staining in all cases. In six of seven cases, loose fibroblastic plugs filling the alveolar spaces were observed after Masson trichrome staining (Figure 1 C). Alveolar septal thickening was noted in three cases with mild fibrosis and accompanying mixed inflammatory infiltrate. Septal capillaries were found to be congested in all cases while pulmonary microthrombi were identified in two cases. Liver tissue showed normal lobular architecture as evidenced by the reticulin framework. Macrovesicular steatosis was seen in seven cases (mild in three, moderate in three, and marked in one) (Table 3 and Figure 1 D). Four cases among these also showed centrizonal congestion with loss of hepatocytes. Portal tracts revealed a mild degree of chronic inflammation in three cases.

**Table 3:** Detailed morphological findings of COVID-19 patients *Steatosis: mild (10-33%), moderate (34-66%), and marked (≥67%) DAD: Diffuse alveolar damage, ASM: Alveolar squamous metaplasia, CA: Corpora amylacea, CZ: Centrizonal, IHC: Immunohistochemistry, MNG: Multinucleated giant cells, NA: Not available, PanZ: Panzonal, Pul. edema: Pulmonary edema, TEM: Transmission electron microscopy, Type II PH: Type II pneumocyte hyperplasia

Patient #	1	2	3	4	5	6	7	8
Lung pathology								
DAD, exudative	+	+	+	+	+	NA	+	+
DAD, organizing	+	+	+	-	+	NA	+	+
Septal thickening	-	+	-	-	-	NA	+	+
Other lung pathology	ASM, MNG, Type II PH, Pul edema, macrophage collection	Type II PH, MNG, microthrombi, macrophage collection	Type II PH, MNG, microthrombi, macrophage collection	-	ASM, Type II PH, MNG, macrophage collection	-	Type II PH, MNG, Pul edema, macrophage collection	Type II, PH
Liver findings								
Steatosis^*^	Moderate, CZ	-	Moderate, CZ	Moderate, CZ	Mild, CZ	Mild, CZ	Mild, CZ	Marked, PanZ
Centrizonal necrosis	-	+	-	+	+	-	-	+
Portal pathology	Mild inflammation	Mild inflammation	-	-	-	-	-	Mild inflammation
Other liver pathology	Mild cholestasis, sinusoidal congestion with neutrophils	Sinusoidal microthrombi, sinusoidal congestion with neutrophils, Kupffer cells hyperplasia	Pericellular fibrosis, mild cholestasis	Sinusoidal microthrombi, sinusoidal congestion with neutrophils	Lobular necroinflammation (neutrophilic), sinusoidal congestion with neutrophils	Sinusoidal congestion with neutrophils, kupffer cells hyperplasia	-	Sinusoidal congestion with neutrophils
Brain findings	CA	CA	CA	CA, hypoxic neurons	CA, hypoxic neurons	CA, old healed infarct	CA	CA, hypoxic neurons
SARS-CoV-2 IHC								
Lung	+	+	-	-	+	NA	-	+
Liver	-	-	-	-	-	-	-	-
Brain	-	-	-	-	-	-	-	-
TEM (viral particles in the lung)	+	+	-	-	NA	NA	-	+

In the brain, corpora amylacea was observed in all cases while occasional hypoxic neurons were seen in three cases. One case showed a focus on an old healed infarct. No intra-parenchymal hemorrhage or perivascular/parenchymal inflammation was noted.

Immunohistochemistry for SARS-CoV-2 nucleocapsid protein

**Figure 2:**
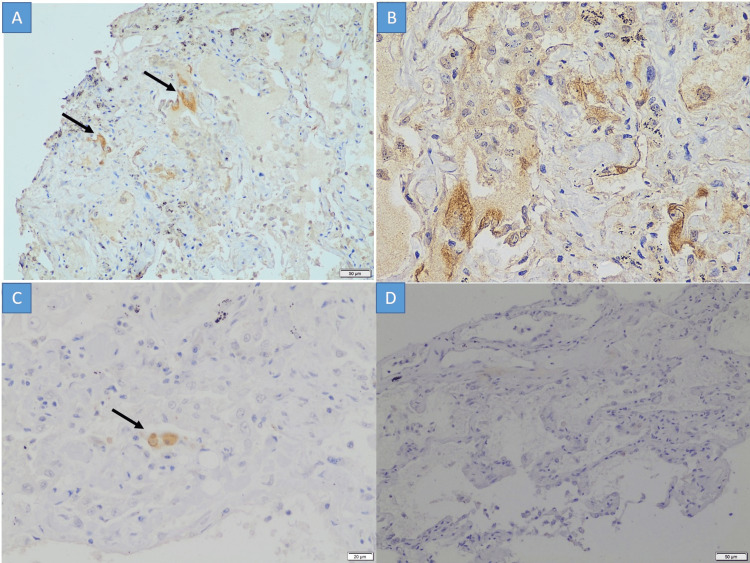
Immunohistochemistry A to C: The SARS-CoV-2 nucleocapsid immunohistochemistry shows intracytoplasmic granular positivity in the alveolar epithelial cells (A x100; B & C x400); D: A SARS-CoV-2 negative case stained with the nucleocapsid immunohistochemistry (D x100)

The IHC positivity for nucleocapsid protein of the SARS-CoV-2 virus was observed in the cytoplasm of alveolar epithelial cells of pulmonary parenchyma in five cases (Figure 2 A to C). The negative control was a lung biopsy taken from a routine surgical pathology sample and showed no reactivity to the SARS-CoV-2 antibody (Figure 2 D). None of the cases had positivity in the liver or brain tissue. 

Transmission electron microscopy findings

**Figure 3:**
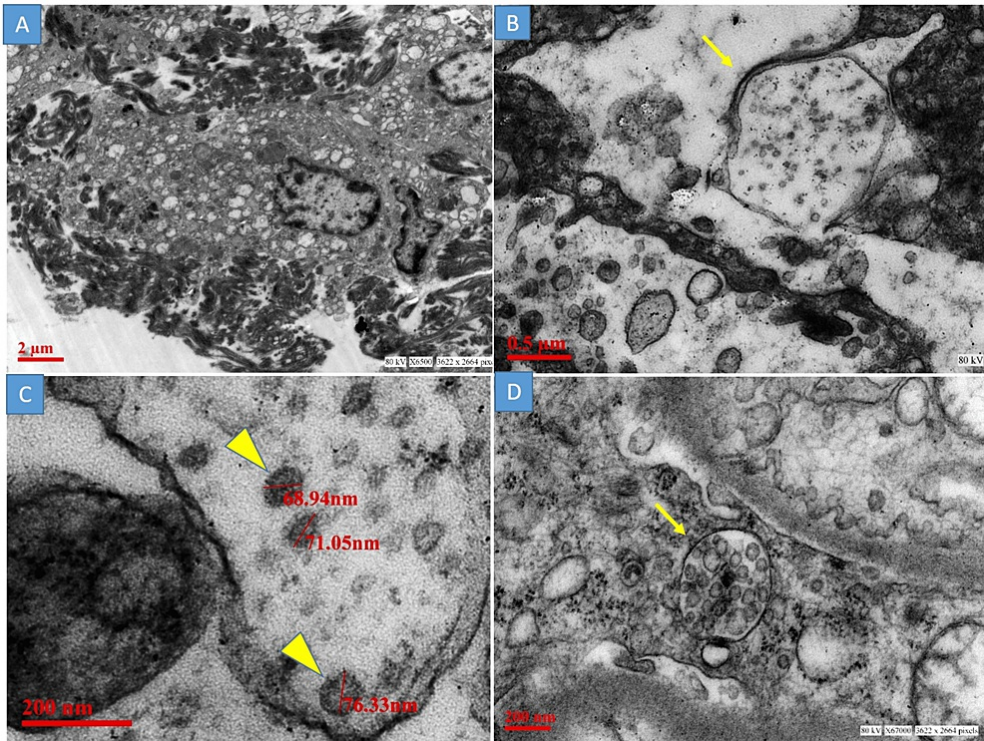
Transmission electron microscopy A: Type II pneumocyte with surface fibrin material; B: Viral particles were observed in double membrane-bound vesicles (yellow arrow), C: Aggregates of round enveloped viral particles of size 68nm to 80nm. Note a few electron-dense dots corresponding to transversely cut helical nucleocapsid (yellow arrowhead); D: A multivesicular body mimicking the viral particles (yellow arrow)

The TEM examination revealed the presence of viral particles in three cases and it was restricted to the lung parenchyma. Type II pneumocytes were identified by the presence of presurfactant secretory granules. These cells were surrounded by electron-dense fibrin threads (Figure 3 A), confirming the findings of the hyaline membrane seen in light microscopy. Viral particles were observed in the vicinity of these cells as small aggregates of round enveloped particles of uniform size 68nm to 80nm. These viral particles were seen intracellularly within double membrane-bound vesicles. Some of these viral particles show the presence of electron-dense dots, corresponding to the helical nucleocapsid (Figure 3 B & C). Ultrastructures mimicking viral particles, including multivesicular body and transversely cut rough endoplasmic reticulum, were also encountered (Figure 3 D). Apart from the lung tissue, no viral particles were noted from the liver and brain tissue.

## Discussion

Several studies have documented the histological changes in various organs in autopsy studies. A few have identified the viral particles on TEM. However, most of these studies are performed outside India. Also, in the existing literature, there is a dispute over the direct extra-pulmonary localization of the coronavirus particles. Further, no ultrastructural studies have been carried out to identify viral particles in autopsy specimens in India. In the present study, we identified the viral particles in the lungs using TEM and IHC. The current study supports the notion that the histology and ultrastructural changes in the lung correlated with the presence of viral particles. We also document that there is an absence of viral particles in the liver and brain tissue.

The present study reports the presence of viral particles and changes in the ultrastructure in cells from the lung, liver, and brain from six cases of SARS-CoV-2 infection. Histopathological findings documented in the present study are similar to those described in Middle East respiratory syndrome coronavirus (MERS-CoV) and severe acute respiratory syndrome coronavirus (SARS-CoV) infections, and recently published reports of SARS-CoV-2 infection [14-18,31,32].

The present study is the first from India to use TEM to assess the ultrastructure of different tissues in SARS-CoV-2 infected patients. The result showed the presence of viral particles in the lung parenchyma, while the brain and liver did not harbor viral particles. Earlier studies using infected cell lines, organoids, and autopsies have reported morphological features of assembled virions and associated cytopathic changes [33,34]. The viral particles have been documented in pulmonary [14,16,35-38] as well as extrapulmonary organs including the kidney [14,16,38-41], heart [38], brain [42], liver [43], intestine [7,44], placenta [45-48] and skin [49]. Most of these studies performed a TEM study in one case [37,39,40,42,44,45,47-49] or in two cases [14,16,38]. Our study reports TEM findings from six cases. Within lung parenchyma, the pneumocytes (both type 1 and type II), bronchial epithelial cells, and endothelial cells are shown to harbor viral particles on TEM examination [14,16,35-38]. The SARS-CoV-2 viral particles were observed in the alveolar respiratory epithelial cells in the present study. Large airway epithelium was not sampled in the present study, hence viral particles in the ciliated respiratory epithelial cells could not be commented on. It has been well established in the literature that viral infection of the host cells is mediated by the binding of virion spike glycoprotein S to its host receptor angiotensin-converting enzyme 2 (ACE2) [50]. The SARS-CoV-2 infection leads to perturbed surfactant production and fluid resorption, resulting in microvascular leakage, ultimately leading to diffuse alveolar damage (DAD) histologically and acute respiratory distress syndrome (ARDS) clinically. We observed the presence of DAD, exudative and organizing phase, as the main pathological findings in the lungs.

Apart from the lungs, viral particles were identified in the kidney tissue in a few studies [14,16,38-40], however, several authors raised serious concerns over these viral particles morphologically consistent with SARS-CoV-2 [51-53]. Dittmayer et al. pointed out that approximately 30 publications in the current literature failed to identify ultrastructural features of SARS-CoV-2 or lacked sufficient image-standard [53]. Goldsmith et al [52]., highlighted the difficulty in identifying viral particles on TEM. Several subcellular structures can be misinterpreted as SARS-CoV-2 viral particles on TEM. These subcellular structures included transversely cut rough endoplasmic reticulum, clathrin-coated vesicles, multivesicular bodies, Golgi vesicles, and glycocalyceal bodies [51]. Correct identification of viral particles relies on the identification of certain morphological features, including intracellular viruses inside double membrane-bound vacuolar structures where viral nucleocapsids appear as small electron-dense dots (6-12nm) often inside the viral particle rather than on the surface, and the size of the viruses (60nm to 140nm) [51,54]. Though spikes are readily visible on tannic acid preparation, they appear as short fuzz on conventional TEM preparation. We could not find viral particles in any samples of liver and brain tissue. This was supported by other studies, where authors could not identify viral particles in these organs [16,37,55].

In the present study, SARS-CoV-2 IHC was positive in the alveolar pneumocytes, while no IHC expression was noted in hepatocytes and neural tissue. Our results are in confirmation with Rocha et al. who reported positive immunohistochemistry staining of SARS-CoV-2 antibodies in the lungs and placenta, while no expression was observed in kidney tissue [56]. Bradley et al. performed TEM, IHC, and RT-PCR in a subset of 14 fatal COVID-19 cases [16]. However, all three approaches for identifying viral particles were conducted in a single patient only. This case had positive IHC expression of SARS-CoV-2 spike protein in the lungs, trachea, and kidney, and viral particle was documented on ultrastructural examination in the lung, trachea, kidney, and large intestine. The same case had SARS-CoV-2 RNA identified in the lung, trachea, subcarinal lymph node, kidney, liver, heart, large intestine, and spleen. Martines et al. performed IHC in eight cases of fatal COVID-19 illness and showed SARS-CoV-2 IHC expression in the lung parenchyma while they could not detect SARS-CoV-2 IHC expression in extrapulmonary organs [57]. Our findings are in concordance with this study. Yao et al. described a single case where coronavirus particles (on TEM) and SARS-CoV-2 nucleocapsid (on IHC) were identified in lung tissue, but no viral presence was detected in the liver, heart, skin, intestine, and bone marrow in either approach [37]. While almost all studies document the presence of direct SARS-CoV-2 infection in pulmonary tissue, there is mixed evidence for the definite viral particles in extrapulmonary organs. The histomorphological changes observed in extrapulmonary organs can be attributed either to the systemic effects of the inflammation or to the direct viral insult. The present study, using light microscopy, IHC and TEM has systematically proved the presence of SARS-CoV-2 virus in the lungs and has provided evidence for morphological changes in the lungs of COVID patients leading to death.

The present study has several limitations. A complete autopsy was not undertaken due to safety limitations. The limited samples obtained using core biopsies during this study may be linked to false negative results in some cases. Another limitation was the small number of patients included in the study. Further studies may reveal that the consistent findings in this cohort are borne out in a large number of tissue samples.

## Conclusions

The specific tropism for SARS-CoV-2 viral particles in the respiratory system is demonstrated using transmission electron microscopy and restricted expression of SARS-CoV-2 nucleocapsid protein using immunohistochemistry in the lung. No viral particles in the liver and brain tissue were identified. Morphological changes observed in these organs may be attributed to the systemic effects of the inflammation.
